# A higher protein intake at breakfast and lunch is associated with a higher total daily protein intake in older adults: a post‐hoc cross‐sectional analysis of four randomised controlled trials

**DOI:** 10.1111/jhn.12838

**Published:** 2020-11-15

**Authors:** A. M. Verreijen, J. van den Helder, M. T. Streppel, I. Rotteveel, D. Heman, C. van Dronkelaar, R. G. Memelink, M. F. Engberink, M. Visser, M. Tieland, P. J. M. Weijs

**Affiliations:** ^1^ Faculty of Sports and Nutrition Center of Expertise Urban Vitality Amsterdam University of Applied Sciences Amsterdam The Netherlands; ^2^ Amsterdam Public Health Research Institute Amsterdam The Netherlands; ^3^ Department of Health Sciences Faculty of Science Vrije Universiteit Amsterdam The Netherlands; ^4^ Department of Nutrition and Dietetics Amsterdam University Medical Centers Vrije Universiteit Amsterdam The Netherlands

**Keywords:** breakfast, dietary protein intake, lunch, older adults, sarcopenia, satiety

## Abstract

**Background:**

A protein intake of 30‐40 g per meal is suggested to maximally stimulate muscle protein synthesis in older adults and could therefore contribute to the prevention of sarcopenia. Protein intake at breakfast and lunch is often low and offers a great opportunity to improve daily protein intake. Protein, however, is known for its satiating effects. Therefore, we explored the association between the amount of protein intake at breakfast and lunch and total daily protein intake in older adults.

**Methods:**

Protein intake was assessed by a 3‐day food record in 498 community dwelling older adults (≥55 years) participating different lifestyle interventions. Linear mixed model analysis was used to examine the association between protein intake at breakfast or lunch and total daily protein intake, adjusted for sex, age, body mass index, smoking status, study and total energy intake.

**Results:**

After adjustment for potential confounders, a 10 g higher protein intake at breakfast was associated with a 3.2 g higher total daily protein intake (*P* = 0.008) for males and a 4.9 g (*P* < 0.001) higher total daily protein intake for females. A 10 g higher protein intake at lunch was associated with a 3.7 g higher total daily protein intake (*P* < 0.001) for males, and a 5.8 g higher total daily protein intake (*P* < 0.001) for females.

**Conclusions:**

A higher protein intake at breakfast and lunch is associated with a higher total daily protein intake in community dwelling older adults. Stimulating a higher protein intake at breakfast and lunch might represent a promising nutritional strategy to optimise the amount of protein per meal without compromising total daily protein intake.

## Graphical abstract

A higher protein intake at breakfast and lunch is associated with a higher total daily protein intake in community dwelling older adults. This association holds true for animal protein, although not for plant protein for which no association was observed. In all, stimulating a higher protein intake at breakfast and lunch might represent a promising nutritional strategy for optimising the amount of protein per meal without compromising total daily protein intake.

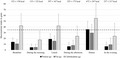

## Introduction

Our society is ageing rapidly ^(^ ^1^ ^)^. Ageing is associated with loss of muscle mass, strength and performance, a process termed sarcopenia. Sarcopenia is associated with an increased risk of falls and fractures, morbidity and mortality. To prevent or even counteract sarcopenia is of major importance because it declines the risk for adverse health outcomes and health‐related cost and improves quality of life ^(^ ^2^ ^)^. The cause of sarcopenia is multifactorial and includes physical inactivity and lower protein intakes ^(^ ^3^ ^)^. Increasing dietary protein intake has been suggested as important beneficial strategy for preventing and/or treat sarcopenia in older adults ^(^ ^4^, ^5^ ^)^.

Phillips *et al*. ^(^ ^6^ ^)^ suggested that a dietary protein intake per meal of 0.4 – 0.6 g kg body weight^−1^ (BW) or approximately 30‐40 g is necessary to maximally stimulate skeletal muscle protein synthesis in older adults. Most community dwelling older adults in the Netherlands do not reach these suggested amounts of protein per meal, particularly at breakfast and lunch: mean (SD) protein intake is 11 ± 7 g at breakfast and 18 ± 10 g at lunch ^(^ ^7^ ^)^. Multiple researchers suggest that an even distribution of proteins over the three meals (and therefore higher protein intakes at breakfast and lunch) with sufficient amounts of protein per meal could translate into a higher anabolic response ^(^ ^8^, ^9^, ^10^, ^11^ ^)^. Kim *et al*. ^(^ ^10^ ^)^ concluded that probably the most efficient way of maximising the anabolic response is to increase dietary protein intake at breakfast and lunch, without reducing protein intake at dinner (for consumption patterns with the hot meal in the evening). Because protein intake at breakfast and lunch in older adults is low ^(^ ^7^ ^)^, these meals offer great potential to increase daily protein intakes ^(^ ^12^ ^)^, aiming to stimulate muscle protein synthesis and optimise muscle maintenance ^(^ ^13^, ^14^ ^)^.

Proteins, however, have a strong satiating effect ^(^ ^15^ ^)^. Increasing the intake in one meal may result in a compensation of protein intakes and other nutrients and energy at other meals ^(^ ^16^ ^)^. This compensation may be influenced by ageing because ageing affects hunger and satiety hormone secretion, as well as feelings of hunger and fullness ^(^ ^17^ ^)^. However, the relationship between protein at breakfast or lunch and total daily protein intake in older adults is unclear ^(^ ^18^, ^19^ ^)^. Therefore, the present study aimed to explore the association between the amount of protein intake at breakfast and at lunch and total daily protein intake in community dwelling older adults.

## Materials and methods

### Study design and study population

A cross‐sectional analysis was performed on baseline data of older adults (≥55 years) participating one of four different lifestyle interventions in the Amsterdam Nutritional Assessment Center at Amsterdam University of Applied Sciences. The four lifestyle interventions were:

1. The MPS (Muscle Preservation Study) ^(^ ^20^ ^)^: a randomised controlled trial in which the effect of a high whey protein‐, leucine‐ and vitamin D‐enriched supplement was tested during a 13‐week weight loss programme including resistance exercise on preservation of muscle mass in an older (≥ 55 years) obese adults. Obesity was defined as a body mass index (BMI) ≥ 30 kg m^–2^ or as a BMI ≥ 28 kg m^–2^ with waist circumference > 88 cm (women) or > 102 cm (men).
2. The WelPrex (Weight Loss with Protein and Exercise) study ^(^ ^21^ ^)^: a randomised controlled trial in which the effect of a high protein diet and/or three times per week resistance exercise was tested during a 10‐week weight loss programme in older (≥ 55 years) overweight and obese adults. Overweight was defined as a BMI ≥ 28 or as a BMI > 25 kg m^–2^ with waist circumference > 88 cm (women) or > 102 cm (men).
3. The PROBE (protein and lifestyle intervention to preserve muscle mass in obese older type 2 diabetes patients) study ^(^ ^22^ ^)^: a randomised controlled trial comparable to the MPS, a 13‐week weight loss trial including resistance training in which the effect of the same supplement was tested, although this population was a diabetic older (≥55 years and older) population with obesity. Obesity was defined as a BMI ≥ 30 kg m^–2^ or as a BMI ≥ 27 kg m^–2^ with waist circumference > 88 cm (women) or > 102 cm (men).
4. The VITAMIN (VITal AMsterdam older adults IN the city) study ^(^ ^23^ ^)^: a randomised controlled trial that evaluated the effectiveness of a digitally supported home‐based exercise training programme, as well as the additional value of dietary protein on physical performance, in community dwelling older adults aged ≥ 55 years.

A full description of the eligibility criteria is available online in the Dutch Trial Register (MPS: NL2623; WelPrex: NL4434; PROBE: NL4357; VITAMINE: NL5472; http://www.trialregister.nl). Written informed consent was obtained from all subjects and the studies were performed in accordance with the Declaration of Helsinki. These studies took place from March 2011 to September 2018 in the Amsterdam Nutritional Assessment Center at the Amsterdam University of Applied Sciences, Amsterdam, The Netherlands.

### Assessment of dietary intake

Baseline dietary intake was assessed by a 3‐day food record at 2 week days and 1 weekend day. Food records were prestructured for the following eating moments: breakfast, in between breakfast and lunch, lunch, in between lunch and dinner, dinner, and in the evening. Subjects were asked to report their food intake as specific as possible and to report amounts of their intake in standard household measures (e.g. three slices of whole grain bread) or to weigh their food items on a kitchen weighing scale. Food records were checked for completeness during study visits by trained fourth grade students Nutrition and Dietetics under supervision of the study dietician. Additional information about unclear items or amounts was obtained and recorded. Food record data of the four studies were collected and verified in accordance with the standard operating procedures of our laboratory. The food items were coded and the nutritional intake data file was coupled to the computerised Dutch Food Composition Table ^(^ ^24^, ^25^ ^)^ to calculate total energy and macronutrient intakes. The dietician or coordinating investigator performed an additional verification and consistency check after the coding process. Subjects with completed dietary records on at least 2 days, and with average reported energy intake of at least 800 kcal day^−1^ were included for analysis. The outcome variable total daily protein intake was calculated in g, g kg BW^−1^ and g kg fat free mass (FFM)^−1^. Protein intake in g kg BW^−1^ was also adjusted for body weight for subjects with a BMI ≥ 30 kg m^−2^ using body weight at BMI 27.5 kg m^−2^^(^ ^26^ ^)^ and for subjects with a BMI < 22 kg m^−2^ using body weight at BMI 22 kg m^−2^^(^ ^27^ ^)^. This adjustment of body weight is applied to make it more comparable to true protein needs and to make it more comparable to that often used in dietetic practice because body composition parameters are not always available. FFM in obese subjects is low relative to their body weight and therefore using actual body weight would probably overestimate protein needs. The opposite is the case for subjects with a low BMI: then, FFM is relatively high for their body weight, and using actual body weight would probably underestimate true needs.

### Assessment of general characteristics and potential confounders

Body composition, including fat mass (FM) and FFM, was determined using air displacement plethysmography (BODPOD, Life Measurement Inc., Concord, CA, USA). Body weight was measured on the calibrated scale as part of the BODPOD system. Body height was measured to the nearest 0.5 cm using a wall‐mounted stadiometer (Seca 222; Seca, Hamburg, Germany). Waist circumference was measured in a standing position halfway between the anterior superior iliac spine and the lower rib after normal expiration (Seca 201; Seca). General characteristics (gender, age and smoking status (current smoker yes or no) were self‐reported at baseline.

### Statistical analysis

Linear mixed model analysis was used to examine the association of protein intake at breakfast (g) and protein intake at lunch (g) with total daily protein intake (g, g kg BW^−1^, g kg adjusted BW^−1^, g kg FFM^−1^) at 2 or 3 days, with a random intercept for subject and a random slope for protein intake at breakfast. The random intercept takes into account that subjects provide dietary intake data from multiple days. The random slope is a variance parameter that is estimated from the different slopes, which is included in the model. These models are adjusted for sex, age, BMI, smoking status, study and total energy intake (kcal day^−1^). Additionally, the association of protein intake at breakfast and protein intake at lunch (g) with protein intake during the rest of the day [total daily protein intake minus protein intake at breakfast or lunch (g)] and protein intake during subsequent meals was studied. Finally, the association of intake of protein source (animal or plant) at breakfast and lunch with total daily protein intake was studied using the same mixed model analysis, with models for animal protein additionally adjusted for plant protein and vice versa.

Effect modification by sex, age, BMI and study was tested for the association between protein intake at breakfast (g) or protein intake at lunch (g) and total daily protein intake (g, g kg BW^−1^, g kg adjusted BW^−1^, g kg FFM^−1^). For most associations sex was an effect modifier; therefore, all analyses were stratified for sex. All analyses were performed using IBM SPSS Statistics for Windows, version 24.0 (IBM Corp., Armonk, NY, USA).

## Results

### Subjects

**Figure 1:**
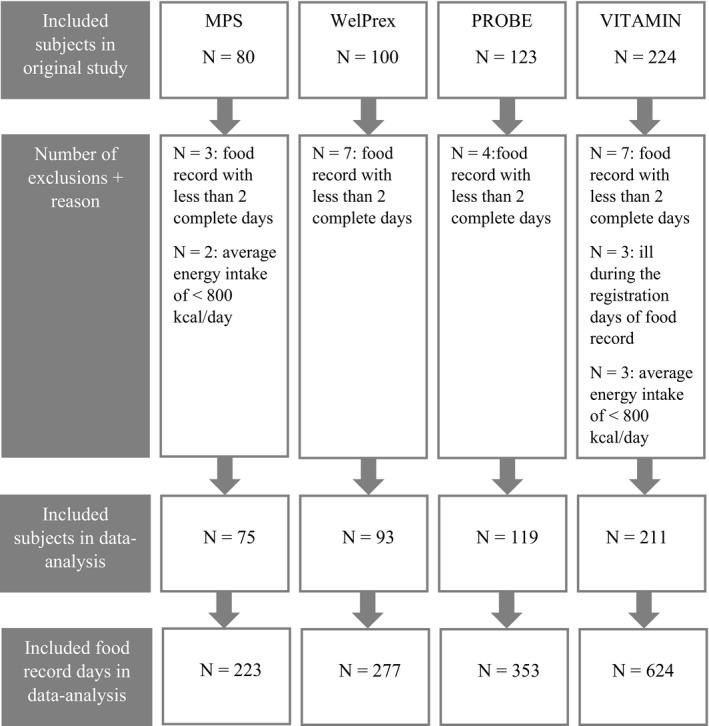
Flow chart for inclusion of baseline data of older adults (*n* = 498) participating in lifestyle interventions at the Amsterdam Nutritional Assessment Center in the data analysis.

In total, 498 participants were included into this analysis. Figure 1 shows the number of participants originally included in each study ^(^ ^20^, ^21^, ^22^, ^23^ ^)^ and the number of food records days used for this analysis. In total 1477 food record days were included in the analysis. The mean (SD) age of the study population was 67.7 (7.3) years, 42% were male; mean BMI was 30.0 (5.6) kg m^−2^ and 21% were normal weight (BMI 20–25 kg m^−2^), 30% were overweight (BMI 25–30 kg m^−2^) and 49% were obese (BMI ≥ 30 kg m^−2^). The general characteristics of the study population are presented in Table 1.

**Table 1:** Baseline characteristics of older adults participating in lifestyle interventions at the Amsterdam Nutritional Assessment Center

	Total study population (*n* = 498)		MPS* (*n* = 75)	WelPrex^*^ (*n* = 93)	PROBE^*^ (*n* = 119)	VITAMIN* (*n* = 211)	*P*‐value^†^
	Mean ± SD/%	Range^‡^	Mean ± SD/%	Mean ± SD/%	Mean ± SD/%	Mean ± SD/%	
Age (years)	67.7 ± 7.3	55–91	63 ± 6	63 ± 5	67 ± 6	72 ± 6	<0.001
% females	58.2%		60.0%	62.4%	33.6%	69.7%	<0.001
Body weight (kg)	86.9 ± 18.5	46.0–146.3	95.4 ± 13.9	92.3 ± 14.5	100.6 ± 15.7	73.7 ± 13.9	<0.001
Height (m)	1.70 ± 0.09	1.50–1.94	1.69 ± 0.09	1.69 ± 0.09	1.73 ± 0.09	1.68 ± 0.09	<0.001
BMI (kg m^−2^)	30.0 ± 5.6	17.5–54.6	33.2 ± 4.4	32.1 ± 4.3	33.6 ± 4.4	25.9 ± 4.2	<0.001
% Overweight^§^	30.3%		24.0%	33.3%	18.5%	37.9%	0.001
% Obese^§^	48.8%		76.0%	65.6%	81.5%	13.3%	<0.001
Waist circumference (cm)	103 ± 15^¶^	66–146	111 ± 11	108 ± 12^††^	115 ± 10^‡‡^	90 ± 11	<0.001
Fat free mass (kg)	51.5 ± 11.9^¶^	28.2–85.3	54.0 ± 10.8**	52.4 ± 12.1^††^	58.5 ± 11.0	46.0 ± 10.0^§§^	<0.001
Fat mass (kg)	35.2 ± 12.2^¶^	9.5–91.3	41.1 ± 10.9**	39.8 ± 9.8^††^	40.6 ± 11.6	27.7 ± 10.0^§§^	<0.001
Body fat percentage (%)	40.0 ± 9.1^¶^	12.6–66.1	43.1 ± 8.6**	43.3 ± 8.4^††^	40.2 ± 8.2	37.2 ± 9.3^§§^	<0.001
% Smoking	7.3%^¶^		9.5%**	8.6%	10.1%	4.3%^§§^	0.180

* The four lifestyle interventions with trial register numbers are the MPS (Muscle Preservation Study): NL2623; the WelPrex (Weight Loss with Protein and Exercise) study: NL4434; the PROBE (protein and lifestyle intervention to preserve muscle mass in obese older type 2 diabetes patients) study: NL4357 and the VITAMIN (VITal AMsterdam older adults IN the city): NL5472 (http://www.trialregister.nl).

^†^ *P*‐value for differences between the four lifestyle interventions. For nominal variables, Pearson’s chi‐squared test is used; for continuous variables, one‐way analysis of variance is used.

^‡^ Range is presented as a minimum to maximum value.

^§^ Overweight = body mass index (BMI) ≥ 25 and < 30 kg m^−2^, obese = BMI ≥ 30 kg m^−2^.

^¶^ *n* waist circumference and *n* smoking status = 495, *n* fat free mass, fat mass and body fat percentage = 479.

** MPS: *n* fat free mass, fat mass and body fat percentage = 70, *n* smoking status = 74.

^††^ WelPrex study: *n* fat free mass, fat mass and body fat percentage and waist circumference = 92.

^‡‡^ PROBE study: *n* waist circumference = 117.

^§§^ VITAMIN study: *n* fat free mass, fat mass and body fat percentage = 198, *n* smoking status = 209.

### Dietary intake

**Figure 2:**
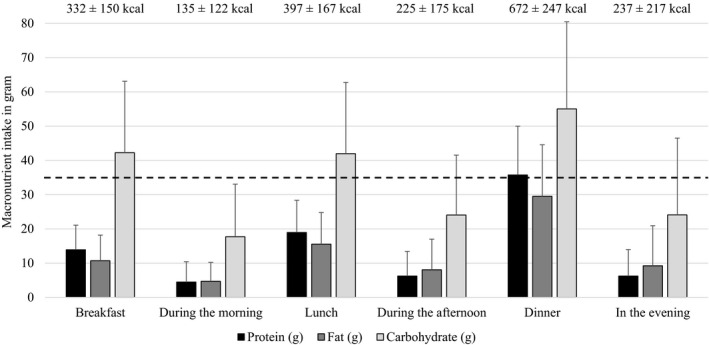
Macronutrient intake per meal. The bars represent an average macronutrient intake per eating moment over the 3‐day food records (*n* = 498). The dashed line represents the amount of protein per meal that is suggested to stimulate protein synthesis^(^ ^28^ ^)^, as calculated using the average body weight of the study population.

Mean (SD) energy intake for the total study population was 1898 (526) kcal, with a protein intake of 82 (24) g or 0.97 (0.30) g kg BW^−1^. Absolute intake of energy and protein was higher for males than for females, whereas protein intake in g kg BW^−1^ day^−1^ and in g kg FFM^−1^ day^−1^ was higher in females (Table 2). In total 70% of the study population reached a protein intake of 0.8 g kg BW^−1^ and 19% reached a protein intake of 1.2 g kg BW^−1^. Only 1% (*n* = 4) reached the suggested amount of 0.4 g kg BW protein^−1^^(^ ^28^ ^)^ at breakfast, with 8% and 51% reaching this value at lunch and dinner, respectively. These percentages are higher using adjusted body weight for subjects with a BMI ≥30 kg m^−2^ or <22 kg m^−2^ and all percentages were higher for females compared to males (Table 2). Figure 2 shows the protein and other macronutrient intakes at all eating moments during the day for the total study population. For males and females, the distribution of protein intake over the day was comparable. For males, mean (SD) protein intake was 15.2 (8.2) g at breakfast, 19.9 (10.3) g at lunch and 38.3 (15.5) g at diner. For females, the intakes were 13.0 (6.2), 18.2 (8.7) and 33.9 (13.0) g, respectively.

**Table 2:** Average dietary intake per day* of older adults participating in lifestyle interventions at the Amsterdam Nutritional Assessment Center

	Total study population *n* = 498		Males *n* = 208	Females *n* = 290
	Mean ± SD, or %	Range^†^	Mean ± SD	Mean ± SD
Energy (kcal)	1898 ± 526	800–4069	2021 ± 521	1810 ± 512
Energy (kJ)	7958 ± 2200	3356–17073	8473 ± 2181	7589 ± 2142
Total protein intake (g day^−1^)	82 ± 24	25–215	88 ± 27	77 ± 23
Plant protein intake (g day^−1^)	29 ± 10	8–72	31 ± 11	28 ± 9
Animal protein intake (g day^−1^)	52 ± 20	5–155	56 ± 20	50 ± 20
Fat intake (g day^−1^)	74 ± 28	15–196	78 ± 27	71 ± 29
Carbohydrate intake (g day^−1^)	195 ± 62	51–443	206 ± 64	186 ± 59
Protein intake energy%	17.6 ± 3.6	8.6–33.4	17.7 ± 3.4	17.5 ± 3.8
Fat intake energy%	34.6 ± 6.8	13.3–59.0	34.4 ± 6.5	34.8 ± 7.0
Carbohydrate intake energy%	41.2 ± 7.3	19.0–75.6	40.9 ± 6.9	41.5 ± 7.6
Protein intake (g kg BW^−1^ day^−1^)	0.97 ± 0.30	0.30–2.33	0.93 ± 0.27	0.99 ± 0.31
Protein intake (g kg adj^‡^ BW^−1^ day^−1^)	1.07 ± 0.31	0.37–2.40	1.04 ± 0.28	1.09 ± 0.32
Protein intake (g kg FFM^−1^ ^§^ day^−1^)	1.64 ± 0.52	0.55–4.29	1.41 ± 0.37	1.81 ± 0.54
% with intake ≥ 0.8 g kg BW^−1^ day^−1^	70%		67%	73%
% with intake ≥ 1.2 g kg BW^−1^ day^−1^	19%		15%	21%
% with intake ≥ 0.8 g/kg adj^‡^ BW^−1^ day^−1^	83%		81%	85%
% with intake ≥ 1.2 g kg adj^‡^ BW^−1^ day^−1^	29%		27%	31%
% consuming ≥ 0.4 g kg BW^−1^ at breakfast	1%		0%	1%
% consuming ≥ 0.4 g kg BW^−1^ at lunch	8%		7%	10%
% consuming ≥ 0.4 g kg BW^−1^ at dinner	51%		46%	56%
% consuming ≥ 0.4 g kg adj^‡^ BW^−1^ at breakfast	2%		1%	2%
% consuming ≥ 0.4 g kg adj^‡^ BW^−1^ at lunch	10%		9%	10%
% consuming ≥ 0.4 g kg adj^‡^ BW^−1^ at dinner	63%		60%	65%

* Average dietary intake is calculated from the mean intake per day of each subject (*n*= 498).

^†^ Range is presented as a minimum to maximum value.

^‡^ Using adjusted body weight for obese subjects [using body weight at body mass index (BMI) 27.5 kg m^−1^] ^(^ ^23^ ^)^ and for subjects with a BMI < 22 kg m^−2^ (using body weight at BMI 22 kg m^−2^) ^(^ ^24^ ^)^.

^§^ Fat free mass (FFM) assessed using air displacement plethysmography (BODPOD, Life Measurement Inc.), *n* total study population = 479, *n* female = 277, *n* male = 202.

The within‐subject coefficient of variation was 23% for total daily protein intake (g), 32% for protein intake at breakfast (g) and 46% for protein intake at lunch (g).

### Association of protein intake at breakfast and lunch with total daily protein intake

Table 3 shows the association of protein intake at breakfast and lunch with total daily protein intake, as well as with protein intake during the rest of the day, adjusted for sex, age, BMI, smoking status, study and total energy intake.

**Table 3:** Associations* of protein intake at breakfast and lunch (g day^−1^) with total daily protein intake, and with protein intake during the rest of the day^†^ and subsequent meals^‡^ in older adults

	Males (*n* = 208)			Females (*n* = 290)		
	Beta	95% CI	*P*‐value	Beta	95% CI	*P*‐value
*Associations of protein intake at breakfast in g day* ^−1^ *(independent variable)*						
Total protein intake (g day^−1^) (dependent variable)						
Crude model^§^	0.90	0.59–1.20	<0.001	1.09	0.82–1.36	<0.001
Adjusted model^§^	0.32	0.09–0.56	0.007	0.49	0.27–0.70	<0.001
Total protein intake (g kg body weight^−1^ day^−1^) (dependent variable)						
Crude model^§^	0.007	0.004–0.010	<0.001	0.010	0.007–0.013	<0.001
Adjusted model^§^	0.002	0.000–0.005	0.048	0.006	0.003–0.009	<0.001
Total protein intake (g kg adjusted body weight^−1^ day^−1^) (dependent variable)						
Crude model^§^	0.009	0.005–0.012	<0.001	0.015	0.011–0.018	<0.001
Adjusted model^§^	0.003	0.000–0.006	0.045	0.007	0.004–0.010	<0.001
Total protein intake (g kg FFM^−1^ ^¶^ day^−1^) (dependent variable)						
Crude model^§^	0.012	0.007–0.016	<0.001	0.021	0.014–0.028	<0.001
Adjusted model^§^	0.004	−0.000 – 0.007	0.068	0.011	0.005–0.016	<0.001
Protein intake during the rest of the day (g day^−1^)^†^ (dependent variable)						
Crude model^§^	−0.10	−0.41–0.20	0.497	0.09	−0.18 – 0.36	0.496
Adjusted model^§^	−0.68	−0.91 – −0.45	<0.001	−0.51	−0.73 – −0.30	<0.001
Protein intake at lunch (g day^−1^) (dependent variable)						
Crude model^§^	−0.08	−0.19 – 0.04	0.191	−0.00	−0.13 – 0.13	0.952
Adjusted model^§^	−0.19	−0.30 – −0.08	0.001	−0.06	−0.19 – 0.08	0.412
Protein intake at dinner (g day^−1^) (dependent variable)						
Crude model^§^	0.12	−0.15 – 0.39	0.397	0.21	0.00 – 0.41	0.048
Adjusted model^§^	−0.08	−0.28 – 0.13	0.462	−0.12	−0.30 – 0.07	0.228
*Associations of protein intake at lunch in g day* ^−1^ *(independent variable)*						
Total protein intake (g day^−1^) (dependent variable)						
Crude model^§^	0.78	0.60–0.96	<0.001	0.98	0.81–1.15	<0.001
Adjusted model^§^	0.37	0.24–0.51	<0.001	0.58	0.46–0.70	<0.001
Total protein intake (g kg body weight^−1^ day^−1^) (dependent variable)						
Crude model^§^	0.007	0.005–0.009	<0.001	0.012	0.010–0.015	<0.001
Adjusted model^§^	0.003	0.002–0.005	<0.001	0.008	0.006–0.009	<0.001
Total protein intake (g kg adjusted body weight^−1^ day^−1^) (dependent variable)						
Crude model^§^	0.009	0.006–0.011	<0.001	0.013	0.011–0.016	<0.001
Adjusted model^§^	0.004	0.002–0.006	<0.001	0.008	0.006–0.009	<0.001
Total protein intake (g kg FFM^−1^ ^¶^ day^−1^) (dependent variable)						
Crude model^§^	0.011	0.008–0.014	<0.001	0.023	0.019–0.028	<0.001
Adjusted model^§^	0.005	0.003–0.007	<0.001	0.014	0.011–0.016	<0.001
Protein intake during the rest of the day (g day^−1^)^†^ (dependent variable)						
Crude model^§^	−0.22	−0.40 – −0.04	0.020	−0.02	−0.19 – 0.15	0.817
Adjusted model^§^	−0.63	−0.76 – −0.49	<0.001	−0.42	−0.54 – −0.30	<0.001
Protein intake at dinner (g day^−1^) (dependent variable)						
Crude model^§^	−0.00	−0.13 – 0.13	0.968	0.14	−0.00 – 0.28	0.054
Adjusted model^§^	−0.19	−0.32 – −0.06	0.005	−0.10	−0.20 – 0.01	0.074

CI, confidence interval.

* For associations with independent variable protein intake at breakfast: analysed with linear mixed models with a random intercept for subject and a random slope for protein intake at breakfast, *n* = 1477 food record days; for associations with independent variable protein intake at lunch: analysed with linear mixed models with a random intercept for subject and a random slope for protein intake at lunch, *n* = 1477 food record days.

^†^ For associations with independent variable protein intake at breakfast: protein during the rest of the day (g) = daily protein intake (g) – protein intake at breakfast (g); for associations with independent variable protein intake at lunch: protein during the rest of the day (g) = daily protein intake (g) – protein intake at lunch (g).

^‡^ For associations with independent variable protein intake at breakfast: subsequent meals are lunch and dinner; for associations with independent variable protein intake at lunch: subsequent meal is dinner.

^§^ The crude model is the model without adjustments; the adjusted model adjusted for sex, age, body mass index, smoking status (current smoker, yes/no), study and total energy intake.

^¶^ Fat free mass (FFM) is assessed using air displacement plethysmography (BODPOD, Life Measurement Inc.), *n* = 1420 food record days.

After adjustment for these potential confounders, a 10 g higher protein intake at breakfast was associated with a 3.2 g higher total daily protein intake (*P* = 0.007) corresponding to a higher total daily protein intake for males of 0.02 g kg BW^−1^ (*P* = 0.048) or 0.03 g kg adjusted BW^−1^ (*P* = 0.045). These associations were stronger for females: a 10 g higher protein intake at breakfast was associated with a 4.9 g higher total daily protein intake (*P* < 0.001) corresponding to a higher total daily protein intake of 0.06 g kg BW^−1^ (*P* < 0.001) or 0.07 g kg adjusted BW^−1^ (*P* < 0.001) (Table 3). However, after adjustment for potential confounders, protein intake at breakfast was significantly negatively associated with protein intake during the rest of the day (total daily protein intake minus protein intake at breakfast): a 10 g higher protein at breakfast was associated with a 6.8 g and 5.1 g lower protein intake during the rest of the day for males and females, respectively. Thus, a 10 g higher protein intake at breakfast did not translate into a 10 g higher total daily protein intake, instead translating into a 3.2 g (males) and 4.9 g (females) higher total intake and therefore a 6.8 g (males) and 5.2 g (females) lower protein intake during the rest of the day (Table 3). A higher protein intake at breakfast was negatively associated with the protein intake at lunch only for males (Table 3). For protein intake at lunch, these associations are in line with the associations for breakfast (Table 3).

When analysing the association of intake of protein source (animal or plant) at breakfast and lunch with total daily protein intake, it appears that this association for plant and animal protein is different. A 10‐g higher animal protein intake at breakfast is associated with a 5.6 g (95% confidence interval = 2.7–8.5 g, *P* < 0.001) higher total daily protein intake for males and a 7.6 g (5.2–1.0 g, *P* < 0.001) higher total daily protein intake for females. A 10 g higher plant protein intake at breakfast, however, is associated with a non‐significant 0.9 g (−2.6–4.3 g, *P* = 0.631) lower total daily protein intake for males and a 2.7 g (−1.0 – 6.5 g, *P* = 0.156) lower intake for females, as well as a significant lower protein intake during the rest of the day, including lunch and dinner. Associations for the source of protein intake at lunch with total daily protein intake, and with protein intake during the rest of the day were in line with the associations described for breakfast.

## Discussion

The present study investigated the association between protein intake at breakfast and lunch with the total daily protein intake among older adults and demonstrates that a higher protein intake at breakfast and lunch is associated with a lower protein intake during the rest of the day (total daily protein intake minus breakfast) but, overall, with a higher total daily protein intake.

In our study population, less than 30% met the suggested recommendation of 1.2 g protein kg BW^−1^^(^ ^29^, ^30^ ^)^ using adjusted body weight ^(^ ^26^, ^27^ ^)^. Having a higher protein intake at breakfast (≥30 g) was associated with more subjects reaching 1.2 g protein kg BW^−1^: 52% versus 28% of the subjects. For lunch, these percentages were 61% versus 25% of the subjects. These findings are in line with the study of Tieland *et al*. ^(^ ^12^ ^)^, in which an even protein distribution over the day, with more protein at breakfast and lunch, was associated a higher percentage of subjects achieving the recommended daily allowance of 0.8 g kg BW^−1^ day^−1^.

Because the present study has a cross‐sectional design, no suggestions for a causal relationship can be made. The study, however, does give an indication that a higher protein intake at breakfast and lunch might have a satiating effect because protein intake at both breakfast and lunch was negatively associated with protein intake during the rest of the day. The total daily protein intake, however, was not compromised and a higher protein intake at breakfast and lunch was still related to a higher total protein intake. However, a higher plant protein intake at breakfast and lunch was not associated with a higher total daily protein intake, in contrast to animal protein. This might suggest that plant protein sources have a stronger satiating effect, although this proposal should be considered with caution because other factors such as the food form also play a role. For example, animal protein might be consumed in more liquid forms (e.g. milk or yoghurt), which probably suppresses appetite less compared to solid forms ^(^ ^31^ ^)^, although this requires further study. Lonnie *et al*. ^(^ ^31^ ^)^ reported that a higher consumption of plant proteins found in whole food also increases dietary fibre, which might amplify satiety. Data regarding the effects of plant proteins on appetite in older adults, however, are very limited and should be investigated in future studies, in addition to the food groups, food form and the food matrix ^(^ ^31^ ^)^.

To our knowledge, the present study is the first to investigate the association between regular protein intake at breakfast and lunch and total daily protein intake. Hengeveld *et al*. ^(^ ^18^ ^)^ demonstrated that older adults (>70 years) with an adequate protein intake (≥0.8 g kg^−1^) had higher protein intakes at all eating occasions, including breakfast and lunch, which is in line with our findings. Several other studies demonstrate that the use of protein enriched meals or foods does not limit and mostly increases the amount of protein per meal and total daily protein intake in older adults ^(^ ^32^, ^33^, ^34^ ^)^. This indicates that satiating effects of higher protein meals or foods are limited in older adults ^(^ ^34^ ^)^. Giezenaar *et al*. ^(^ ^35^ ^)^ showed that although gastric emptying was slower in older compared to younger men, which gives a prolonged post‐prandial satiety, the acute administration of whey protein drinks before a meal suppressed subsequent energy intake in young, but not in healthy older men. These findings were substantiated by Clegg *et al*.^(^ ^36^ ^)^.

Only 2% and 10% of our subjects reached the suggested amount of 0.4 g kg protein^−1^^(^ ^28^ ^)^ at breakfast and lunch, which suggests that habitual protein intake during breakfast and lunch is generally low. The range of habitual protein intake at breakfast and lunch in the present study, however, is large and is achieved with regular food products. This shows that a higher protein intake at breakfast is achievable for some older adults and also demonstrates potential for improvement. A higher protein intake at breakfast and lunch may lead to a higher number of eating occasions that reach the suggested anabolic threshold for optimal muscle protein synthesis ^(^ ^28^ ^)^. Regardless of the total daily protein intake, this is already a potential gain, which might impact subsequent muscle maintenance or accretion ^(^ ^6^ ^)^ and is important with respect to preventing or counteracting sarcopenia. However, this has not yet been substantiated by long‐term dietary intervention trials.

A limitation of the present study is the high percentage of obese older adults (almost 50%) in our study population. Obese adults have a higher prevalence of carrying the specific single nucleotide polymorphisms in the fat mass and obesity‐associated gene (FTO) ^(^ ^37^ ^)^. FTO might facilitate weight gain by decreasing the release of the satiety hormone leptin and increasing the release of hunger‐promoting hormone ghrelin ^(^ ^38^ ^)^. Therefore, the satiating effect of a meal might be lower in obese subjects. The representativeness of the study population compared to the general older population may thus be low. In the present study, however, we did not observe differences in the association of protein intake at breakfast and lunch with total daily protein intake between obese and non‐obese subjects. This suggests that potential differences in the release of hunger and satiety hormones for obese versus non‐obese subjects do not appear to translate into differences in the relationship between protein intake at breakfast or lunch and protein intake during the rest of the day. A lower protein intake at breakfast, however, was related to a lower BMI: the 10% of the participants with the lowest protein intake at breakfast had a significantly lower BMI than subjects with a higher protein intake at breakfast [28.3 (4.8) versus 30.2 (5.6) kg m^−2^]. Because BMI was also related to the primary outcome total daily protein intake, all models were adjusted for BMI. Another limitation concerns the reported energy intake in the present study, which is comparable to that for Dutch older adults in general ^(^ ^18^ ^)^, whereas almost half of our study population was obese. We therefore expected a higher energy intake in our study population. Based on previous research ^(^ ^39^ ^)^, overweight people tend to underestimate their dietary intake more often than normal‐weight people, and therefore true energy and protein intake could be underestimated in our study. Park *et al*. ^(^ ^40^ ^)^ demonstrated that a dietary food record has advantages compared to a food frequency questionnaire: less under‐reporting of energy and nutrients. In both overweight and obese subjects, protein intake with a dietary record was less under‐reported than energy intake. A third limitation is that we did not adjust for the potential confounding factors education‐level and income ^(^ ^41^ ^)^ because these variables were not available for all included studies. A final limitation is that our study population had a wide age range, from 55 to over 90 years. Although age was no effect modifier in the relationship between protein intake at breakfast or lunch and total daily protein intake, the dietary intake of food groups and the dietary pattern may change during the ageing process as a result of a wide variety of factors ^(^ ^42^ ^)^.

The present study also has some strengths. We used a 3‐day dietary food record to assess protein intake, which probably gives a more realistic estimate of dietary intake than a recall‐method in this older population because it is likely to be less prone to short‐term memory loss. In addition, we used a linear mixed model analysis that took into account the within‐subject, day‐by‐day variation of dietary intake, which provides a more sensitive analysis than using an average dietary intake per subject.

## Conclusions and implications

In conclusion, a higher protein intake at breakfast and lunch is associated with a higher total daily protein intake in community dwelling older adults. This association holds true for animal protein, although not for plant protein for which no association was observed. In sum, stimulating a higher protein intake at breakfast and lunch might represent a promising nutritional strategy for optimising the amount of protein per meal without compromising total daily protein intake.

## Transparency declaration

The lead author affirms that this manuscript is an honest, accurate and transparent account of the study being reported. The reporting of this work is compliant with STROBE guidelines. The lead author affirms that no important aspects of the study have been omitted and that any discrepancies from the study as planned have been explained.
